# Liquid Biopsy-Derived Tumor Biomarkers for Clinical Applications in Glioblastoma

**DOI:** 10.3390/biom15050658

**Published:** 2025-05-02

**Authors:** Bruna Pereira de Lima, Leticia Silva Ferraz, Sylvie Devalle, Helena Lobo Borges

**Affiliations:** 1 Instituto de Ciências Biomédicas, Universidade Federal do Rio de Janeiro (UFRJ), Av. Carlos Chagas Filho, 373, Rio de Janeiro 21941–902, RJ, Brazil; pereira.bruna2000@gmail.com; 2 Centro de Ciências Naturais e Humanas (CCNH), Universidade Federal do ABC (UFABC), Av. dos Estados, 5001, Santo André 09280-560, SP, Brazil; leticia.conconi@ufabc.edu.br; 3 Instituto Estadual do Cérebro Paulo Niemeyer (IECPN), Rua do Rezende, 156-Centro, Rio de Janeiro 20231-092, RJ, Brazil; devallelbmc@gmail.com

**Keywords:** liquid biopsy, glioblastoma, tumor biomarkers, circulating tumor cells, circulating tumor cells, circulating tumor DNA, circulating free RNA, extracellular vesicles

## Abstract

Glioblastoma (GBM) is the most aggressive primary brain tumor in adults, characterized by rapid growth and resistance to chemotherapy. Conventional treatments remain largely ineffective, with patient survival averaging around 18 months after diagnosis. Current diagnostic methods rely on invasive tissue biopsies and imaging tests. While traditional biopsies involve extracting tissue samples, their routine use is often limited by surgical risks and the challenge of accessing sensitive brain regions. Liquid biopsy has emerged as a promising noninvasive alternative, analyzing circulating tumor components—such as DNA, RNA, extracellular vesicles, and microRNAs—found in body fluids. This approach enables initial diagnosis and continuous disease monitoring, offering a significant advantage over traditional biopsies, which are impractical for frequent repetition during treatment follow-up. This review highlights recent advances in liquid biopsy-derived biomarkers for the clinical management of GBM. The discussion includes the advantages, limitations, and potential of these biomarkers as tools for early diagnosis and disease monitoring. A narrative review of the literature published over the last decade (2014–2024) was conducted using major health-focused scientific databases. The analysis focuses on evaluating the clinical relevance and applicability of liquid biopsy in GBM, offering insights into its potential as a minimally invasive and effective tool for improving glioblastoma management.

## 1. Introduction

### 1.1. Glioblastoma Overview

Glioblastoma (GBM) is the most aggressive and prevalent primary brain tumor in adults, with a poor prognosis and a median survival of 14 to 20 months after diagnosis, even with aggressive multimodal treatments [1]. GBM is highly invasive and characterized by rapid proliferation and resistance to conventional therapies, including surgery, radiation, and chemotherapy [2]. One of the main challenges in treating GBM is its intrinsic heterogeneity, both within the tumor (molecular and cellular levels) and between patients, leading to variable responses to treatment and frequent therapeutic resistance [3]. This heterogeneity complicates the development of universal therapeutic strategies, as distinct tumor regions can evolve independently and foster diverse resistance mechanisms.

Histopathological and molecular analysis of tissue obtained via biopsy or resection remains the gold standard for GBM diagnosis. Additionally, surgery is necessary to relieve mass effect, delay recurrence, and enable molecular profiling [1]. Current clinical methods for monitoring GBM progression rely primarily on imaging techniques, such as computed tomography (CT) and magnetic resonance imaging (MRI). However, these approaches have limitations, particularly in distinguishing true tumor progression from pseudoprogression, a phenomenon where treatment-induced tissue changes, such as necrosis and inflammation, mimic tumor growth but do not represent actual tumor activity [4]. In contrast, true progression involves active tumor proliferation and growth. Advanced techniques, such as perfusion MRI and PET scans, may provide more specific insights into tissue dynamics [5], but are not broadly available in the clinical setting. Additionally, liquid biopsy biomarkers, including circulating tumor DNA (cfDNA) and extracellular vesicles (EVs), show promise in overcoming these limitations by offering real-time, minimally invasive tools to monitor tumor progression and therapeutic response [6], as well as in distinguishing true progression from pseudoprogression [7]. These advancements highlight the potential to improve GBM management, where current methods remain insufficient [8].

In this context, liquid biopsy has emerged as a promising method with reduced invasiveness for capturing tumor-derived biomarkers in body fluids like blood, plasma, or cerebrospinal fluid (CSF). Liquid biopsy analyzes tumor-derived biomarkers, including extracellular vesicles (EVs), such as exosomes, which carry DNA, RNA, and proteins that reflect the molecular characteristics of the tumor [9]. Liquid biopsy enables the detection of tumor-derived molecules circulating in body fluids, offering a minimally invasive strategy that may complement imaging and tissue biopsies, particularly in the longitudinal monitoring of tumor progression and recurrence. It is hypothesized that liquid biopsy could capture molecular signals from a broader array of tumor regions than localized tissue biopsy, potentially offering insights into spatial and temporal heterogeneity. This may be especially valuable in clinical scenarios where conventional biopsy is limited, such as tumors located in deep or eloquent brain areas.

Moreover, emerging data suggest that certain biomarkers—such as circulating tumor cells, extracellular vesicles, or circulating macrophages—may provide noninvasive molecular information that could support clinical decision-making, particularly when imaging findings are inconclusive [10]. Despite this promise, the approach remains largely investigational due to key limitations, including the presence of the blood–brain barrier (BBB) and the typically low abundance of tumor biomarkers in GBM circulation, which hinder consistent detection. Additionally, while liquid biopsy may offer a route to assess tumor heterogeneity, since analytes from different tumor areas could theoretically reach the bloodstream, this potential still requires robust clinical validation. This remains to be confirmed; however, one study has shown that mutations absent from tumor specimens were detected in the plasma of several glioblastoma patients [11]. Although false-positive cases cannot be ruled out, the fact that patients with mutations detected in plasma displayed reduced survival argues against this hypothesis.

For now, liquid biopsy should be regarded as an investigational and complementary tool to histopathology, which remains the gold standard for diagnosis due to its direct visualization of tumor tissue and established diagnostic accuracy. Even so, the ability of liquid biopsy to access molecular information from otherwise inaccessible tumor regions underscores its promise and fully justifies continued efforts toward its clinical development and integration [12].

### 1.2. Liquid Biopsy Overview

Liquid biopsy refers to the detection and quantification of tumor-derived elements (tumor biomarkers), such as cell-free nucleic acids (DNA/RNA), circulating tumor cells (CTCs), extracellular vesicles (EVs), proteins, and metabolites found in biological fluids [6]. Its primary advantage lies in the ability to provide real-time insights into tumor dynamics, including genetic alterations, tumor burden, clonal fitness or acquisition, and resistance mechanisms, without requiring highly invasive procedures [9]. This capability has positioned liquid biopsy as a key tool in precision oncology, enabling the capture of tumor heterogeneity and the detection of emerging resistant clones, which helps clinicians adapt treatment strategies more effectively [6,13].

In the case of GBM, liquid biopsy offers a less invasive strategy that may complement imaging and tissue biopsies, particularly in longitudinal monitoring of tumor progression and recurrence. While imaging techniques such as amino acid PET and MRI perfusion have demonstrated high sensitivity and specificity in distinguishing true progression from pseudoprogression and can even provide insights into molecular characteristics [14,15], their broader application remains limited by the availability of specialized equipment and trained personnel. Traditional MRI continues to serve as the clinical cornerstone, yet emerging evidence suggests that liquid biopsy markers—such as circulating tumor cells, extracellular vesicles, or circulating macrophages—may offer additional, noninvasive insights that support and enrich current diagnostic approaches [10]. Although still in an investigational stage, liquid biopsy holds strong potential as a valuable adjunct to imaging, contributing complementary molecular information that may enhance the accuracy and timing of clinical decision-making in glioblastoma care [6,10]. Despite challenges posed by the BBB and the typically low levels of cfDNA in the bloodstream, alternative approaches, such as CSF analysis, and the collection of larger blood volumes are being explored [7]. Successful integration of liquid biopsy in GBM could provide valuable insights into tumor evolution and therapeutic responses, ultimately contributing to improved patient outcomes.

## 2. Materials and Methods

This review explores recent advancements and challenges in liquid biopsy for glioblastoma, with a particular focus on technological innovations, clinical applications, and future perspectives. A comprehensive literature search was conducted using renowned scientific databases, including PubMed-MEDLINE, Scopus, Web of Science, and Google Scholar. The keywords used were ‘glioblastoma’, ‘liquid biopsy’, and ‘biomarker’. Articles published within the last 10 years (2014–2024) were selected, specifically those addressing the clinical application of liquid biopsy-derived tumor biomarkers in glioblastoma.

The selection process prioritized studies based on their originality and relevance to glioblastoma and liquid biopsy, with emphasis on research presenting clinical data, technological advancements, or field-specific challenges. Studies focusing on other tumor types or unrelated to liquid biopsy applications were excluded.

Data analysis involved extracting information on recent advances, advantages, limitations, and the clinical potential of biomarkers derived from liquid biopsy in the context of glioblastoma. Any discrepancies in article selection were resolved through discussion and consensus among the reviewers.

## 3. Selected Papers and the Clinical Applicability of Biomarkers Derived from Liquid Biopsy in Glioblastoma

### 3.1. Panorama of the Selected Papers

**Figure 1:**
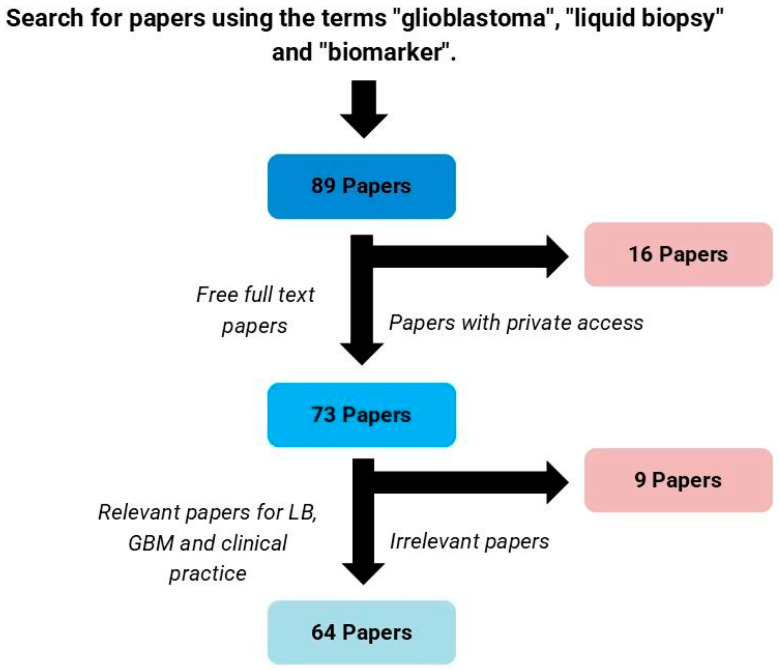
Process for selection of articles. LB: liquid biopsy; GBM: glioblastoma.

When adopting the described methodology, 89 articles were identified, of which only 73 had the full text available for access. After screening titles and abstracts for their meaningful contribution to the liquid biopsy field on GBM, 64 articles were selected for discussion in this review (Figure 1).

The 64 studies were then classified according to the biological matrix used, the sample size, the type of biomarker, and the analysis technique employed (Table 1).

### 3.2. Circulating Tumor Cells (CTCs)

Circulating tumor cells (CTCs) are shed into the bloodstream from primary or metastatic tumors and serve as a valuable tool for tumor characterization when tissue biopsies are not feasible [80]. However, detecting CTCs is challenging due to their rarity, found at approximately one cell per billion blood cells [81]. The quality and quantity of isolated CTC samples can vary depending on the isolation method, impacting the sensitivity and specificity of detection [82].

Standard techniques for CTC detection include qPCR (both DNA and cDNA), although these approaches may generate false positives. The CellSearch^®^ System, validated by the FDA, is a robust method but has limited applicability in glioblastoma because it primarily targets epithelial markers, such as EpCAM, which are often absent in tumors of the central nervous system (CNS), including GBM. Furthermore, the significant cellular heterogeneity in GBM makes single-marker detection difficult [83,84,85].

Advanced methods like the CTC chip and EPISPOT enable the isolation of viable tumor cells for more detailed analyses [86]. Lynch et al. challenged the assumption that brain tumor cells do not cross the BBB by isolating GBM-derived CTCs using immunomagnetic enrichment and identifying specific markers like GLAST to distinguish GBM-CTCs from lymphocytes [17]. Similarly, Bark et al. used microfluidic technology to isolate CTCs from newly diagnosed GBM patients (18/20 IDH wild-type), reporting that patients with zero CTC counts post-surgery experienced longer recurrence-free survival [18].

Kolostova et al. analyzed peripheral blood from GBM patients after tumor resection, finding a high concordance between the molecular profiles of CTCs and their respective primary tumors. Interestingly, they identified more mutations in CTCs than in the primary tumors [19]. Recent findings have shown that CTCs in glioblastoma can display mesenchymal and stem-like features, contributing to their invasive potential and ability to migrate through the bloodstream. Although extracranial metastases are extremely rare in GBM due to the restrictive nature of the brain environment, the blood–brain barrier and the restricted survival time of GBM patients, they have been reported in isolated cases involving organs such as the lungs and bones. Śledzińska et al. highlighted that CTCs may harbor distinct molecular alterations compared to the primary tumor, potentially contributing to metastatic spread. Mutations in *EGFR*, *RB1*, and *SETD2*, for example, have been identified in metastatic sites despite being absent in the corresponding primary tumor [82]. These rare occurrences highlight the complex biology of GBM and underscore the need for further investigation into CTCs and their role in tumor spread. Additionally, Gao et al. reported that CTCs showed potential in monitoring treatment response and distinguishing radionecrosis from glioma recurrence, although the study was limited to a small patient cohort, warranting further validation [16].

In summary, despite the technical challenges associated with CTC isolation and detection, these cells hold significant potential for prognosis and patient monitoring in GBM. They allow molecular assessments of the disease and reveal new aspects, such as distinct GBM cell populations circulating in the bloodstream with potentially unique properties. However, further studies are required to validate and integrate these approaches into clinical practice.

### 3.3. Extracellular Vesicles (EVs)

Extracellular vesicles (EVs) are lipid membrane-bound nanoparticles of various sizes, classified based on their cellular origin and biogenesis mechanisms [87,88]. Exosomes, a specific subtype of EVs, are extensively studied in glioblastoma liquid biopsy due to their abundance in bodily fluids and their ability to transport biomolecules, including genetic and protein material that reflect the tumor’s status and can be useful for diagnosis and monitoring [89,90,91].

Recent studies have emphasized the potential of EVs as tumor biomarkers, particularly when combined with conventional tumor markers. Yang et al. investigated gene expression in brain and blood exosomes using an orthotopic xenograft mouse model. In primary tumors and blood exosomes, DNM3, p65, and CD117 expression increased, while PTEN and p53 decreased (both at the mRNA and protein levels). In recurrent tumors, DNM3 and p65 remained elevated, whereas ST14 and p53 decreased, suggesting that serum exosomes could distinguish primary from recurrent GBM based on gene/protein transcription/expression profiles [20].

Hallal et al. identified 4054 proteins in EVs isolated from plasma samples, among which a subset was found to correlate with glioma histological grades. EVs from aggressive tumors exhibited elevated levels of proteins such as AIDA, BNIP3L, CETN3, FYB1, and POLR2D [21]. Similarly, Cilibrasi et al. highlighted the potential of inflammatory proteins, including VWF, FCGBP, C3, PROS1, and SERPINA1, in EVs derived from IDH wild-type GBM patients, reinforcing their role in disease monitoring [23]. Dobra et al. demonstrated that small extracellular vesicles (sEVs) were more effective than whole serum in differentiating GBM from brain metastasis, meningioma, and disc herniation [22].

Lennartz et al. investigated vesicular heat shock protein 70 (Hsp70) and its effect on the immunophenotypic profile of lymphocytes in glioma patients. Elevated Hsp70 levels in GBM patients were associated with a decrease in CD3+/CD4+ T cells and a worse prognosis [27]. Conversely, in grade 3 gliomas, elevated Hsp70 levels and activated NK cells correlated with better survival. Similar findings on increased Hsp70 levels in GBM were reported by Werner et al. [28].

Nucleic acids, particularly miRNAs and piRNAs, have also been studied as EVs cargo in the liquid biopsy context. MicroRNAs (miRNAs) are small, non-coding RNA molecules (18–25 nucleotides) that regulate gene expression post-transcriptionally by targeting mRNAs for degradation or translational inhibition, while PIWI-interacting RNAs (piRNAs) are longer non-coding RNAs (24–31 nucleotides) primarily involved in silencing transposable elements and maintaining genomic stability, especially in germ cells. Akers et al. profiled EV-derived miRNAs from tumor tissue and CSF, identifying a GBM-specific signature of nine miRNAs, including miR-21, miR-218, and miR-193b. The signature showed higher sensitivity in cisternal CSF compared to lumbar CSF, confirming that proximity to the tumor enhances detection [36]. Shi et al. further demonstrated elevated levels of miR-21 in CSF-derived EVs from glioma patients, with no significant differences in serum-derived EVs [37].

Hallal et al. identified piRNAs, including piR_016658, piR_016659, and piR_020829, in EVs derived from ultrasonic aspirates and serum, distinguishing GBM from lower-grade gliomas and healthy controls [32]. Stella et al. found decreased levels of CircSMARCA5 and circHIPK3—circular RNAs (circRNAs), a class of stable, non-coding RNAs formed by back-splicing, which regulate gene expression and are often dysregulated in cancer—in serum EVs from GBM patients compared to lower-grade gliomas and controls. CircSMARCA5 and circHIPK3 have been implicated in glioma progression and tumorigenesis, highlighting their potential as diagnostic markers [62]. Larger studies have identified additional circRNAs, such as hsa_circ_0055202, hsa_circ_0074920, and hsa_circ_0024108, as promising diagnostic and prognostic markers [65,92].

Genetic alterations detected in EVs have demonstrated significant clinical utility. Rosas-Alonso et al. showed that *MGMT* methylation analysis in EVs correlates with improved responses to temozolomide chemotherapy and better prognostic outcomes, often surpassing tissue biopsies due to sampling limitations [38]. Similarly, Ricklefs et al. reported that elevated EV plasma levels in GBM patients were associated with shorter overall survival (OS) and progression-free survival (PFS), allowing tumor recurrence to be predicted months before MRI detection [30]. Expanding on this, Aibaidula et al. utilized spectral flow cytometry to identify non-neoplastic EVs in GBM patient plasma, highlighting new possibilities for non-invasive biomarker development [29]. Hallal et al. further demonstrated that urine-derived EVs from IDH wild-type GBM patients carried molecular signatures indicative of glioblastoma, representing a promising, fully non-invasive approach for tumor detection and monitoring [24]. However, limitations such as small cohort sizes and the absence of validated common EV markers for GBM underscore the need for further research. In conclusion, although several studies suggest the promise of EV-based liquid biopsy in the context of GBM, its clinical applicability for disease monitoring has yet to be firmly established.

### 3.4. Circulating Tumor Nucleic Acids (ctNAs)

Circulating tumor DNA (ctDNA) has emerged as a promising biomarker in GBM liquid biopsy, offering a non-invasive approach to monitor tumor evolution and guide treatment decisions. Despite the low concentration of ctDNA in the plasma of glioma patients, advances in next-generation sequencing (NGS) and PCR platforms have significantly improved its detection and clinical relevance. CtDNA can be identified through molecular markers such as mutations, rearrangements, copy number variations, epigenetic modifications, and abnormal fragmentation patterns. Mattos-Arruda et al. demonstrated that CSF-derived ctDNA is more representative of brain tumor genomic alterations than plasma ctDNA, making it particularly useful for guiding treatment in recurrent GBM cases [45]. Similarly, Miller et al. detected CSF ctDNA in 49.4% of cases, correlating its presence with disease burden and adverse outcomes. The genetic alterations identified in CSF closely mirrored those found in tumor biopsies, including mutations in IDH1/2 and the 1p/19q codeletion, facilitating the development of targeted therapies for glioma [40]. Although IDH1/2-mutated tumors and those with 1p/19q codeletion are no longer classified as GBM, these findings highlight the potential of CSF for detecting tumor-related DNA alterations and improving differential diagnosis.

Dai et al. established CSF ctDNA methylation profiles in recurrent GBM patients using data from the China glioma genome atlas (CGGA) and the Gene expression omnibus (GEO). Using Lasso and Cox multiplex analyses, they identified eight core genes (*FLRT2*, *NKD1*, *GNB5*, *NTRK3*, *COMMD1*, *C1orf226*, *CHI3L2*, and *ETV1*) for diagnostic and prognostic models, demonstrating high predictive accuracy [48]. Bagley et al. investigated the association between cfDNA concentration and survival in GBM patients, finding that higher preoperative cfDNA levels correlated with shorter progression-free survival (PFS) and overall survival (OS). Moreover, an increase in cfDNA concentration following chemoradiotherapy, compared to preoperative baseline levels, was associated with worse survival projections, reinforcing its potential as a prognostic biomarker [46].

Juratli et al. evaluated the detection of *TERT* promoter (*TERT*p) mutations in ctDNA from CSF and plasma in GBM patients, detecting mutations in 38 of 60 cases. The sensitivity of *TERT*p mutation detection in CSF (92.1%) was significantly higher than in plasma (7.9%). Additionally, a higher variant allele frequency (VAF) of *TERT*p mutations in CSF was linked to poor survival, indicating its potential as a prognostic biomarker [41].

Meng et al. explored magnetic resonance imaging-guided focused ultrasound (MRgFUS) as a non-invasive technique to improve access to circulating brain biomarkers by temporarily disrupting the BBB. Their study demonstrated that MRgFUS, combined with temozolomide (TMZ) treatment, effectively increased plasma levels of cfDNA, neuron-derived EVs, and the glial injury marker S100b, highlighting its potential in enhancing liquid biopsy sensitivity for central nervous system tumors [47].

Zhao et al. analyzed ctDNA mutations in various glioma subtypes, finding a high concordance between CSF ctDNA mutations and tumor DNA mutations in 17 paired samples. Mutations in *PTEN* and *TP53* were frequently detected in recurrent gliomas, while IDH1/2 mutations were predominant in astrocytomas, and *RB1* and *EGFR* mutations were identified in IDH wild-type GBM. These results underscore the potential of CSF ctDNA in distinguishing glioma subtypes [42]. These observations were supported by Miller et al., who demonstrated that CSF ctDNA is useful for monitoring tumor evolution and serves as a valuable prognostic tool [40]. Contrary to most studies, Piccioni et al. detected ctDNA alterations in 55% of GBM patients (n = 222) using the Guardant360 assay, with significant implications for personalized targeted therapy and treatment monitoring [44].

Cell-free RNAs (cfRNAs) are another important class of circulating biomarkers, released into biological fluids through passive secretion from necrotic or apoptotic cells, as well as active secretion via membranous vesicles. These molecules include messenger RNAs (mRNAs) and non-coding RNAs (ncRNAs), the latter subdivided into small (e.g., microRNAs, miRNAs) and long (lncRNAs) forms. Studies indicate that while less than 2% of the human genome is transcribed into protein-coding RNAs, the majority of the transcriptome consists of ncRNAs, increasing interest in their viability as cancer biomarkers [12,93].

MiRNAs play a crucial role in gene expression regulation and are actively released into the CSF and bloodstream, acting as both biomarkers and therapeutic targets in GBM. They influence tumorigenesis and cancer progression, affecting key processes such as cell proliferation, invasion, and resistance to treatment. Several studies have linked miRNA profiling to disease staging, prognosis, and treatment response assessment [6,94].

Lucero et al. identified eight miRNAs (miR-148a-3p, miR-9-5p, miR-9-3p, miR-22-3p, miR-182-5p, miR-186-5p, miR-16-2-3p, and miR-378e) associated with angiogenesis and poor survival in endothelial cells exposed to glioblastoma stem cell-derived EVs (GSC-EVs). Notably, silencing miR-148a normalized tumor vasculature in murine GBM models, suggesting that miRNA export by GSCs may contribute to therapy resistance [49].

Drusco et al. identified a CSF miRNA signature that differentiates GBM from other CNS malignancies. Their analysis revealed upregulation of miR-451, miR-223, and miR-125b in GBM compared to normal individuals, miR-711 upregulation in GBM versus lymphoma and medulloblastoma, and downregulation of miR-125b in GBM versus medulloblastoma. Notably, GBM lacks miR-935 expression, distinguishing it from other CNS tumors [50]. These findings provide insights into GBM molecular characteristics, aiding diagnosis and potentially informing treatment strategies.

Additional studies support miRNA expression as a diagnostic and prognostic tool. D’Urso et al. identified increased miR-21 and miR-15b levels in glioma patient serum, with miR-16 downregulation distinguishing GBM from lower-grade gliomas [52]. Qu et al. also observed higher miR-21 levels in CSF than in tumor tissue, reinforcing its value as a prognostic indicator [51]. Lai et al. reported that serum miRNA-210 levels were significantly elevated in GBM patients, correlating with tumor grade and poor outcomes [56]. Other studies demonstrated that serum miR-221 and miR-222 levels were associated with poor survival [60], while miR-100 upregulation post-treatment correlated with improved prognosis [57].

LncRNAs have also been implicated in GBM. Amer et al. identified increased lncRNA565 and lncRNA641 expression in serum samples, correlating with worse PFS and OS, suggesting their role as prognostic biomarkers and therapeutic targets [64]. Chen et al. identified lncRNA MALAT1 as a prognostic marker, with increased expression correlating with TMZ resistance and reduced survival [66].

Taken together, these findings underscore the growing potential of ctDNA, cfRNAs, and extracellular vesicle-derived markers in glioblastoma liquid biopsy. While ctDNA in CSF has demonstrated greater sensitivity and informativeness for brain tumor analysis, ctDNA detection in peripheral blood—although less invasive—still lacks sufficient sensitivity for clinical application. Nonetheless, circulating biomarkers may offer a promising avenue for future approaches aiming to classify gliomas, capture tumor heterogeneity, inform treatment strategies, and support disease monitoring within the framework of precision oncology. As research continues, addressing challenges related to detection sensitivity, standardization, and clinical validation will be crucial to fully integrate liquid biopsy into routine GBM management. Moreover, despite advances in the process of CSF acquisition, careful consideration regarding the benefits of CSF-based liquid biopsy is needed given the risks associated with repetitive lumbar puncture [95].

### 3.5. Circulating Proteins and Metabolites

Metabolomics encompasses the investigation of small molecules present in biofluids, cells, tissues, or organs, enabling the study of metabolic pathways within the organism [96]. Metabolites represent the final products of protein translation and gene transcription, as well as cellular modifications affecting the proteome, genome, and transcriptome. The metabolome serves as the interface between the external environment and the genome, comprising bioactive small molecules such as nucleotides, carbohydrates, amino acids, and fatty acids. Identifying metabolites and alterations in metabolic pathways is crucial for understanding disease pathophysiology and discovering potential therapeutic targets [97]. Additionally, circulating proteins in biofluids provide a broader molecular profile of tumor heterogeneity. These proteins often include membrane receptors, receptor ligands, growth factors, and cytokines. Given the relatively low cost of isolation techniques, these approaches have been widely explored [98,99]. Research involving these proteins primarily focuses on evaluating their altered levels in tumor cells due to the lack of glioma-specific proteins [12]. Consequently, circulating proteins and metabolites are emerging as promising tumor biomarkers derived from liquid biopsy. These components reflect not only the molecular characteristics of the tumor but also its interactions with the surrounding microenvironment [94,95]. The integration of proteomic and metabolic data with genomic and clinical information has the potential to enhance the identification of predictive, prognostic, and therapeutic response biomarkers, thus advancing precision medicine [100].

Dufrusine et al. demonstrated that galectin-3, encoded by the *LGALS3BP* gene, is overexpressed in EVs derived from the serum of GBM patients and correlates with tumor volume. Their findings suggest that tumor cells increase the release of EVs expressing *LGALS3BP*, making galectin-3 a promising biomarker for diagnosis and disease monitoring [26]. Likewise, Masood et al. reported that both protein and mRNA levels of PD-L1 were elevated in GBM patients’ plasma, with high levels correlating with poor overall survival, suggesting its potential as a prognostic marker and therapeutic target [68]. Soler et al. identified that the ratio between CD14+ blood cells with low levels or no HLA-DR ( HLA-DR^neg/low^) and those positive for VNN2 (VNN2⁺) can differentiate patients with recurrent GBM from those with radiation necrosis, highlighting its potential in challenging clinical cases [70]. Ghorbani et al. investigated four potential biomarkers—glial fibrillary acidic protein (GFAP), neurofilament light (NfL), matrix metalloproteinase 3 (MMP3), and fatty acid-binding protein type 4 (FABP4)—as non-invasive markers for glioblastoma. Their study suggested that these proteins, detectable in blood samples, could aid in GBM diagnosis and patient monitoring. However, they were unable to differentiate GBM from lymphomas [71].

Bark et al. applied metabolomics to analyze saliva and blood samples from 21 newly diagnosed GBM patients, 20 of whom were IDH wild-type. Among 151 metabolites and 197 lipids identified, cyclic AMP (adenosine monophosphate), 3-hydroxykynurenine, dihydroorotate, UDP (uridine diphosphate), and cis-aconitate were increased, while oxamic acid was decreased in patients with poor outcomes. Additionally, lipid profiles in these patients exhibited greater heterogeneity and fewer marker associations compared to patients with better prognosis [77]. Shen et al., in a larger cohort of newly diagnosed GBM patients, found that decreased plasma levels of arginine and methionine as well as increased levels of kynurenate were associated with poorer two-year OS and PFS [75]. Liu et al. conducted a pilot study on the metabolomic characterization of human glioblastomas and patient plasma samples to identify metabolic profiles suitable for diagnosis and monitoring. Their results revealed significant metabolic differences between GBM patients and healthy individuals, suggesting that metabolomics could provide valuable insights into tumor status and disease progression. The study proposes that metabolomics could enhance liquid biopsy approaches, improving early diagnosis and enabling personalized treatment strategies [76].

Further studies have demonstrated the potential of plasma metabolomics to classify glioma patients with high accuracy. Zhao et al. showed that metabolomic profiling could distinguish low- and high-grade gliomas with 91.1% accuracy using a panel of 18 metabolites and differentiate IDH-mutated from IDH wild-type patients through six specific metabolites. Notably, lower plasma levels of arginine (indicating increased tumor uptake) in high-grade gliomas suggest that patients could be stratified for arginine-deprivation therapy. Additionally, uridine and ornithine plasma levels could be used to distinguish GBM from malignant gliomas, aiding differential diagnosis [74]. Furthermore, 2-hydroxyglutarate (2-HG) has been proposed as a key metabolic marker for IDH-mutant gliomas [101], although these are now categorized separately from GBM according to the 2021 WHO classification. Björkblom et al. found that high serum levels of vitamin E in grade 4 glioma patients could predict disease years before diagnosis, aligning with studies linking elevated vitamin E levels to an increased risk of developing certain cancers and its role in tumor progression [72]. Supporting this, a study by Bao et al. also reported a significant association between elevated alpha-tocopherol levels and glioblastoma risk [73], reinforcing the potential relevance of this antioxidant in glioma pathogenesis. Lipidomics, another growing field, has also provided promising insights. Zhou et al. conducted a large-scale study incorporating machine learning and identified an 11-lipid plasma signature capable of differentiating malignant gliomas from healthy subjects with an accuracy of 0.9641 [78]. Other research groups have reported quantitative differences in various lipid classes, although these findings were based on smaller patient cohorts [79].

These findings underscore the potential of metabolomics, proteomics, and lipidomics in glioblastoma liquid biopsy. Integrating these molecular signatures with genomic and transcriptomic data enhances tumor characterization, enabling earlier diagnosis, personalized treatment, and better patient monitoring. Advancing standardization, expanding cohort studies, and improving technologies will be key to fully implementing these approaches in precision oncology, ultimately refining GBM diagnostics and patient management.

## 4. Technical Challenges and Future Directions

The low concentration of certain biomarkers, such as ctDNA and CTCs, reduces the sensitivity of isolation techniques and the detection of genetic alterations in liquid biopsy analyses. Additionally, biological variability among patients and tumor heterogeneity can influence the quantity and quality of detected biomarkers. Furthermore, biological barriers like the BBB can limit the availability of biomarkers in peripheral blood, further decreasing the sensitivity of these tests [12].

Sensitivity and specificity vary significantly across different studies and techniques. While some studies report high sensitivity and specificity for certain biomarkers, such as ctDNA in CSF, others show more modest results for plasma-derived biomarkers [102]. Mattos-Arruda et al. and Miller et al. demonstrated that CSF-derived ctDNA more accurately represents brain tumor genomic alterations than plasma ctDNA [40,45], suggesting that CSF may be a more suitable choice for liquid biopsy in GBM, particularly for patient follow-up [37]. Having said so, although uncommon, complications related to repeated lumbar punctures [95,103,104,105] might limit their practical use in routine clinical follow-up. Thus, enhancing the sensitivity and robustness of liquid biopsy analyses from peripheral blood—an easier and safer source—would be highly preferable.

Traditional biopsy remains the gold standard for glioblastoma diagnosis, with high diagnostic accuracy ranging from 90–96% in clinical studies [106]. In contrast, liquid biopsy offers a non- or minimally invasive alternative that enables real-time monitoring of disease dynamics, although its diagnostic accuracy is generally lower than that of tissue-based methods. Table 2 summarizes the diagnostic performance of different liquid biopsy strategies in glioblastoma, comparing their sensitivity and specificity to matched tumor tissue. Among the fluids analyzed, CSF consistently outperformed plasma and serum, particularly in ctDNA-based assays, reaching up to 95% sensitivity for TERT promoter mutations. EVs biomarkers also showed encouraging results, with EV-RNA from serum achieving 81.6% sensitivity and EV-miRNA panels from CSF demonstrating specificity as high as 95%. While performance varies depending on the biomarker type, fluid source, and analytical platform, these findings support the role of liquid biopsy—especially CSF-based approaches—as a valuable complementary tool. Its utility may be particularly relevant in cases where traditional biopsy poses increased risk or is not feasible.

In addition, liquid biopsy enables molecular monitoring through the detection of tumor-specific alterations in circulating biomarkers. A 2021 study introduced the GeLB score, a serum-based DNA methylation signature that accurately distinguishes glioma patients from controls (sensitivity: 100%, specificity: 97.8%). Importantly, the GeLB score was able to differentiate true progression from pseudoprogression, decreasing in cases with histologically confirmed pseudoprogression despite suspicious MRI findings [107]. Although highly promising, it is important to note that this study analyzed gliomas of various types together, and the performance of the GeLB score specifically in glioblastoma remains to be confirmed in larger, subtype-specific cohorts.

In a recent paper published in *Clinical Chemistry* (2025), Iorgulescu et al. demonstrated that analyzing larger volumes of plasma using a personalized ctDNA assay called MAESTRO-Pool significantly improves the sensitivity of liquid biopsy for glioblastoma [7]. MAESTRO-Pool is a mutation-enrichment method that uses patient-specific tumor “fingerprints” to selectively capture and sequence rare ctDNA fragments from the bloodstream with high precision. A key finding was the assay’s ability to help differentiate true tumor progression from pseudoprogression, an ongoing clinical challenge in neuro-oncology. In cases where MRI results were indeterminate, ctDNA detection strongly correlated with histologically confirmed true progression, while its absence aligned with cases of pseudoprogression confirmed by pathology. These results suggest that high-yield, mutation-informed liquid biopsy can offer a valuable, noninvasive tool to support clinical decision-making in glioblastoma management by providing early molecular evidence to complement imaging and guide treatment response assessment.

Combining liquid biopsy data with imaging may thus improve diagnostic precision and guide clinical decisions. Among the available techniques, CSF-derived ctDNA analyzed by NGS and ddPCR currently appears to be one of the most promising approaches, offering higher sensitivity and specificity in detecting tumor-specific alterations [40,41,42,44]. Despite these advances, liquid biopsy is better positioned as a complementary tool, particularly useful in cases where surgical procedures present high risks, such as in deep-seated or brainstem lesions, and in therapeutic monitoring, where early detection of molecular changes may precede radiological progression.

Additionally, studies comparing different analytes compartments, such as exosomes, plasma, serum, and whole blood, are necessary to determine whether exosomes offer advantages for biomarker detection. Exosomes are particularly promising due to their abundance in blood, resistance to degradation, and their demonstrated superiority over whole blood in protein-based liquid biopsies [22,26].

A lack of concordance is observed among studies analyzing the same biomarker type (e.g., circulating microRNAs, circular RNAs), which may be attributed to variations in sample size, GBM classification criteria, patient cohorts (e.g., recurrent vs. newly diagnosed, treated vs. treatment-naïve, different therapies), selected biological matrices (e.g., serum vs. extracellular vesicles), and methodological differences in sample isolation and analysis. Indeed, 70% of the studies cited in this review analyzed fewer than 50 patients and employed diverse reagents, isolation methods, analytical platforms, and statistical approaches, often using heterogeneous or poorly defined cohorts. This highlights the urgent need for standardization in liquid biopsy methodologies.

Regarding specificity, the detection of non-tumor-specific genetic alterations, such as clonal hematopoiesis of indeterminate potential (CHIP), can lead to false positives and complicate diagnosis. However, parallel whole-blood analysis can help distinguish true tumor-derived alterations from CHIP-related findings. Currently, eight registered clinical trials (www.clinicaltrial.org, accessed on 15 December 2024) are dedicated to evaluating liquid biopsy in GBM patients, which may help address some of these challenges. The PLANET (NCT05099068) and GRETeL (NCT05695976) trials focus on validating ctDNA as a marker for therapy response, survival, recurrence, and its ability to reflect tumor alterations. Another trial (NCT05934630) is assessing cfDNA in CSF from pediatric and young adult with primary brain tumors. A novel approach involves the collection of tear fluid as a liquid biopsy matrix to evaluate the effects of tumor-treating fields (TTFields) in GBM patients (NCT06136611), which could provide a less invasive alternative for biomarker analysis.

In parallel, researchers are actively developing new techniques to improve access to certain biomarkers [108]. For example, Lynch et al. established an immunomagnetic enrichment method targeting MCAM (melanoma cell adhesion molecule) and MCSP (melanoma-associated chondroitin sulfate proteoglycan), followed by the use of GLAST (glutamate aspartate transporter) and GFAP (glial fibrillary acidic protein) to differentiate CTCs from lymphocytes, thereby improving the identification of GBM-derived CTCs [17].

Another approach to enhance liquid biopsy sensitivity involves increasing BBB permeability, as demonstrated by Meng et al. [47]. Two ongoing clinical trials (NCT05383872, NCT05281731) are currently validating the impact of ultrasound-based transient BBB permeabilization on the detection of GBM ctDNA.

Additionally, advances in detection techniques are further improving the sensitivity and specificity of liquid biopsy-based analyses. Digital droplet PCR (ddPCR) and next-generation sequencing (NGS) methodologies are being refined to enhance the detection of cfDNA and other biomarkers, reducing false negatives. The integration of bioinformatics tools is also significantly improving the accuracy of liquid biopsy-based NGS [109]. These advancements hold great promise for enhancing the early detection and monitoring of glioblastoma.

**Figure 2:**
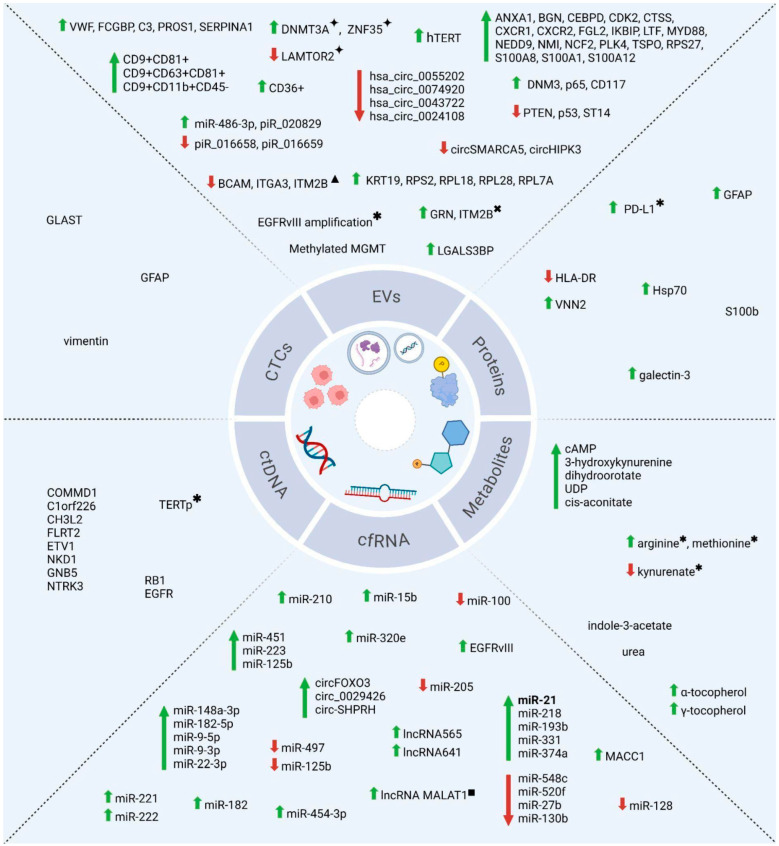
Key liquid biopsy biomarkers for glioblastoma. Schematic overview of main biomarkers discussed in the study. Green arrows indicate increased analyte levels; red arrows indicate decreased levels. Biomarkers shown without arrows were reported as simply detected in GBM liquid biopsy. Biomarkers identified in multiple studies (2014–2024) are highlighted in bold (e.g., miR-21). ✦ in responders to dacomitinib; ✱ in patients with poor survival; ✖ in recurrent patients; ▲ in resected patients; ■ in non-responders to temozolomide. Image created with BioRender (https://BioRender.com/s28x312).

In summary, liquid biopsy represents a promising non-invasive tool for helping the clinical evaluation of glioblastoma. In this review, we highlighted the increasing importance of various biomarkers, including ctDNA, cfRNAs, EVs, CTCs, proteins, and metabolites, in characterizing tumors, detecting mutations, identifying methylation and genome structural alterations, assessing recurrence risk, predicting prognosis, and monitoring therapeutic responses (Table 3, Figure 2).

## 5. Final Remarks

Despite its potential, several challenges remain, particularly the low levels of cfDNA in the bloodstream due to limited tumor DNA release, rapid clearance, and the restrictive nature of the BBB, as well as the invasive nature of CSF collection. However, technological advancements in ddPCR, NGS, multi-omics integration and biomarker enrichment techniques offer promising solutions to enhance detection sensitivity and specificity. Future innovations, including more sensitive assays and less invasive sampling methods, could significantly improve the clinical applicability of liquid biopsy in GBM, ultimately leading to better patient outcomes.

Nonetheless, before liquid biopsy can be fully integrated into clinical practice, rigorously designed clinical trials using standardized methodologies are essential to validate the reproducibility, specificity, and prognostic value of liquid biopsy biomarkers. These trials should follow the WHO 2021 guidelines and involve large, well-defined patient cohorts to ensure clinical reliability.

Although liquid biopsy offers a minimally invasive alternative for obtaining molecular information in GBM, it is important to emphasize that, at present; this method does not achieve the same diagnostic accuracy or therapeutic value as tissue biopsy or surgical resection. In addition, surgery may be required at different stages of the disease for various purposes, including symptom relief, tumor debulking, and molecular profiling. The true promise of liquid biopsy lies in its potential to complement existing tools—rather than replace them—offering a valuable option for continuous disease monitoring and for patients in whom surgical access to the tumor poses significant risk.

In addition, the current concentration of studies on specific analytes, such as vesicular or free ctDNA, miRNAs, and mRNAs, combined with small sample sizes, heterogeneous cohorts, and variations in analytical methods, underscores the urgent need for standardization, careful patient selection, and diversification of biomarkers and biological matrices. While challenges remain, the results so far are promising, and it is expected that at least some liquid biopsy biomarkers will be validated in the near future for GBM diagnosis, monitoring, and personalized treatment strategies. Notably, the field of fragmentomics and strategies such as small fragment enrichment remain unexplored for GBM in this new exciting field and warrants future investigation in the search for more sensitive liquid biopsy approaches.

## Figures and Tables

**Table 1:** Classification of scientific studies included in review. cfDNA: cell-free deoxyribonucleic acid; CGGA: Chinese glioma genome atlas; circRNA: circular ribonucleic acid; CSF: cerebrospinal fluid; CTC: circulating tumor cell; ctDNA: circulating tumor deoxyribonucleic acid; ddPCR: droplet digital polymerase chain reaction; ELISA: enzyme-linked immunosorbent assay; EV: extracellular vesicle; FFPE: formalin-fixed paraffin-embedded; GBM: glioblastoma; IDH: isocitrate dehydrogenase; IHC: immunohistochemistry; LC-MS: liquid chromatography–mass spectrometry; lncRNA: long noncoding ribonucleic acid; mRNA: messenger ribonucleic acid; miRNA: micro ribonucleic acid; NA: not available; NGS: next-generation sequencing; PBMC: peripheral blood mononuclear cell; PCR: polymerase chain reaction; piRNA: PIWI-interacting RNA; qPCR: quantitative (real-time) polymerase chain reaction; qRT-PCR: quantitative reverse-transcribed PCR; WB: western blot; WT: wild-type. * Only IDH wild-type (true GBM according to the WHO 2021 classification) were considered; ^▲^ No information on IDH status or GBM classification version. Summary data based on information from [16,17,18,19,20,21,22,23,24,25,26,27,28,29,30,31,32,33,34,35,36,37,38,39,40,41,42,43,44,45,46,47,48,49,50,51,52,53,54,55,56,57,58,59,60,61,62,63,64,65,66,67,68,69,70,71,72,73,74,75,76,77,78,79].

Authors	Biological Matrix	GBM Sample Size (n)	Biomarker	Method (*Platform of* *Analysis/Assay*)	Ref.
Gao et al., 2016	Blood	11 ^▲^	CTC (enumeration)	SE-iFISH (*Olympus BX-53*)	[16]
Lynch et al., 2020	Blood and cell line	13 ^▲^	CTC (enumeration)	Immunocytostaining (*CellCelector*), flow cytometry *(BD FACS Canto II*)	[17]
Bark et al., 2021	Blood	20 (18 GBM IDH-WT)	CTC (enumeration)	Immunofluorescence (*Zeiss Axio Imager Z2*)	[18]
Kolostova et al., 2021	Blood	18 ^▲^	CTC (DNA)	NGS (*GeneReader*)	[19]
Yang et al., 2017	Plasma and tumor tissue	4 ^▲^	EV (RNA, protein), tissue RNA	Microaaray (*NA*), WB (*NA*)	[20]
Hallal et al., 2020a	Plasma	41 (24 GBM IDH-WT)	EV (protein)	SWATH-MS (*TripleTOF^®^6600/Eskpert^TM^ NanoLC 425*)	[21]
Dobra et al., 2020	Serum	24 ^▲^	EV (protein)	LC-MS (*Q Exactive Plus*)	[22]
Cilibrasi et al., 2022	Plasma	15 *	EV (protein)	Mass spectrometry (*Q Exactive/Dionex Ultimate 3000 RSLCnano*)	[23]
Hallal et al., 2024	Urine	24 *	EV (protein)	Liquid chromatography (*Ultimate 3000*) and mass spectrometry (*Q-Exactive HFX3*)	[24]
Brahmer et al., 2023	Serum and cell line	9 ^▲^	EV (protein)	Multiplex bead-based flow cytometry (*MACSPlex Neuro EV in Attune NxT*)	[25]
Dufrusine et al., 2023	Plasma, serum, tumor tissue, and cell line	17 ^▲^	EV (protein), protein	WB (*NA*), ELISA (*KE00155*), IHC (*NA*)	[26]
Lennartz et al., 2023	Plasma, serum, and tumor cell	99 *	EV (protein), protein	ELISA (*Hsp70-exo*, *R&D Systems DuoSet)*, Multiparameter Flow Cytometry (*BD FACSCalibur*)	[27]
Werner et al., 2021	Serum and cell line	34 ^▲^	EVs, protein	ELISA (*R&D Systems DuoSet),* WB *(NA)*, Flow Cytometry *(BD FACSCalibur*™)	[28]
Aibaidula et al., 2023	Plasma	20 *	EV (surface protein)	Spectral flow cytometry (*Cytek Aurora*)	[29]
Ricklefs et al., 2024	Plasma	101 ^▲^	EV (quantification, surface protein)	Nanoparticle tracking analysis (*NanoSight LM14*), and imaging flow cytometry (IFCM) (*ImageStreamX* *Mark II Imaging*)	[30]
Figueroa et al., 2017	CSF and tumor tissue	55 ^▲^	EV (RNA)	qRT-PCR (*ABI Prism 7500*)	[31]
Hallal et al., 2020b	Surgical aspirate and plasma	17 (12 GBM IDH-WT)	EV (RNA)	NGS (*NextSeq 500*)	[32]
Yekula et al., 2023	Serum	14 ^▲^	EV (RNA)	NGS—RNA-seq (*NextSeq 500*)	[33]
Uziel et al., 2024	Serum	61 (60 GBM IDH-WT)	EV (RNA)	qRT-PCR (*Step One*)	[34]
Manda et al., 2018	Serum and tumor tissue	73 ^▲^	EV (RNA), tissue RNA	End-point RT-PCR *(Master Cycler Pro S)*	[35]
Akers et al., 2017	Tumor tissue andCSF (EV and total)	111 ^▲^	EV (miRNA), miRNA	TaqMan OpenArray^®^ Human MicroRNA Panel (*Taqman OpenArray),* qRT-PCR (*CFX96*)	[36]
Shi et al., 2015	Serum and CSF	45 ^▲^	EVs (miRNA)	qRT-PCR (*NA*)	[37]
Rosas-Alonso et al., 2024	Plasma and FFPE tumor tissue	50 *	EV (DNA), tissue DNA	Quantitative methylation-specific PCR (qMSP) (*NA*)	[38]
Wang et al., 2015	CSF	35 (9 GBM IDH-WT)	ctDNA	whole-exome sequencing (WES), SafeSeqS Pipeline	[39]
Miller et al., 2019	CSF, plasma, and tumor tissue	46 (44 GBM IDH-WT)	ctDNA	NGS *(MSK-IMPACT)*	[40]
Juratli et al., 2018	Tumor tissue, CSF, and Plasma	38 *	ctDNA	NGS (*Ion Torrent PGM*), ddPCR (*QX200*)	[41]
Zhao et al., 2020	Tumor tissue and CSF	4 *	ctDNA	NGS (*Ion Proton*)	[42]
Wang, Q. et al., 2023	CSF and tumor tissue	27 (12 GBM IDH-WT)	cfDNA	NGS (*NovaSeq 6000 system*), IHC (*NA*)	[43]
Piccioni et al., 2019	Plasma	222 ^▲^	ctDNA	NGS (*Guardant360*)	[44]
Mattos-Arruda et al., 2015	CSF, plasma, and tumor tissue	4 ^▲^	ctDNA, tissue DNA	NGS (*HiSeq 2000*), ddPCR (*QX200*)	[45]
Bagley et al., 2021	Plasma	62 *	cfDNA (quantification)	qPCR (*ViiA 7*)	[46]
Meng et al., 2021	Plasma	9 (8 GBM IDH-WT)	cfDNA (quantification and methylation profiling), EV, protein (quantification)	MethylationEPIC 850k array, ddPCR (*QX200*), ELISA (*EZHS100B-33K*)	[47]
Dai et al., 2023	CSF and tumor tissue	4 ^▲^ + 109 *in sillico* (CGGA)	RNA	RNA-seq (*NovaSeq and NovaSeq 6000*)	[48]
Lucero et al., 2020	Human brain endothelial cells (HBMVECs)	Not applicable	miRNA	DNA methylation profiling (*Human 450K Infinium Methylation BeadChip*) and RNA-seq (*NextSeq 500 and HiSeq 4000*), histoepigenetic analyses (*NA*),	[49]
Drusco et al., 2015	CSF	4 ^▲^	miRNA	Microarray (*nCounter NanoString*)	[50]
Qu et al., 2016	CSF and tumor tissue	35 ^▲^	miRNA	qRT-PCR (*NA*)	[51]
D’Urso et al., 2015	Serum and plasma	16 ^▲^	miRNA	qRT-PCR (*7500*), microarray (*Affymetrix 428*)	[52]
Sun et al., 2015	Serum	61 ^▲^	miRNA	qRT-PCR (*NA*)	[53]
Regazzo et al., 2016	Serum	10 ^▲^	miRNA	qRT-PCR *(ABI PRISM 7900)*	[54]
Xiao et al., 2016	Plasma	39 ^▲^	miRNA	qRT-PCR (ABI *PRISM 7300*)	[55]
Lai et al., 2015	Serum	32 ^▲^	miRNA	qRT-PCR (*DNA Engine Opticon 2*)	[56]
Zhang et al., 2019	Serum	95 (67 GBM IDH-WT)	miRNA	qRT-PCR (*ABI PRISM 7900*)	[57]
Morokoff et al., 2020	Serum	44 (29 GBM IDH-WT)	miRNA	Micro array (*nCounter NanoString*), ddPCR (*NA*)	[58]
Yue et al., 2016	Serum	27 ^▲^	miRNA	qRT-PCR (*ABI PRISM 7900*)	[59]
Swellam et al., 2019	Serum	20 ^▲^	miRNA	qRT-PCR (*Max3005P*)	[60]
Shao et al., 2015	Plasma	22 ^▲^	miRNA	qRT-PCR (*ABI PRISM 7500*)	[61]
Stella et al., 2021	Serum and tumor tissue	23 ^▲^	circRNA	ddPCR (*QX200*), qRT-PCR (*ABI PRISM 7900 HT*)	[62]
Chen et al., 2020	Plasma	100 ^▲^	circRNA	qRT-PCR (*NA*)	[63]
Xia et al., 2021	Plasma	120 ^▲^	circRNAs	qRT-PCR, circRNA microarray analysis	[65]
Amer et al., 2022	Serum	35 ^▲^	lncRNA	qRT-PCR (*Max 3005P*)	[64]
Chen et al., 2017	Serum	140 ^▲^	lncRNA	qRT-PCR (*ABI PRISM 7500*)	[66]
Hagemann et al., 2019	Plasma	45 (36 GBM IDH-WT)	mRNA	qRT-PCR (*LightCycler 480*)	[67]
Masood et al., 2023	Plasma	64 ^▲^	mRNA, protein	qRT-PCR (*NA*), ELISA (*Human PD-L1 Platinum*)	[68]
Tsvetkov et al., 2021	Plasma	84 (19 IDH-WT)	Protein	nanoDSF Prometheus NT.Plex instrument (*Nanotemper*)	[69]
Soler et al., 2017	Blood	18 ^▲^	PBMC (surface protein)	Flow cytometry (*BD C6*)	[70]
Ghorbani et al., 2024	Plasma	67 *	Protein	MSD^®^ Electroluminescence multiplexed immunoassays	[71]
Björkblom et al., 2016	Serum	110 ^▲^	Metabolite	Mass spectrometry/chromatography (*Leco Pegasus 4D TOFMS/Agilent 6890*)	[72]
Bao et al., 2024	CSF	91	Metabolite	Mendelian randomization (GWAS-based)	[73]
Zhao et al., 2016	Plasma	18 ^▲^	Metabolite	LC-QQQ-MS (*Sciex 5500 QTRAP/Agilent 1200*)	[74]
Shen et al., 2018	Plasma	159 (105 GBM IDH-WT)	Metabolite	Mass spectrometry (*NA*)	[75]
Liu et al., 2024	Plasma and tumor tissue	15 *	Metabolite	Mass spectrometry (*1290 UHPLC/Sciex TripleTOF 6600 and 1290 UHPLC/Agilent 6530 QTOF and 6550 QTOF mass spectrometer*)	[76]
Bark et al., 2023	Plasma and saliva	21 (20 GBM IDH-WT)	Metabolite, lipid	LC-QqQ-MS (*Agilent* *6470/Infinity II Flex UHPLC*)*,* LC-QTOF-MS (*Agilent 6546/Infinity II Flex UHPLC*)	[77]
Zhou et al., 2022	Serum	377 ^▲^ (139 in the validation cohort)	Lipid	Liquid chromatography/mass spectrometry (*Ultimate 3000/Q-Exactive MS*)	[78]
Soylemez et al., 2023	Blood	14 ^▲^	Lipid	Liquid chromatography/mass spectrometry (*6530 Accurate-Mass Q-TOF LC/MS*)	[79]

**Table 2:** Diagnostic performance of liquid biopsy approaches compared to tissue biopsy in glioblastoma. Table summarizes sensitivity and specificity for each biomarker studied, using tumor tissue as reference when applicable. Summary data based on information from [31,35,36,38,39,40,41,42,44,45].

Biomarker	n	Source	Sensitivity	Specificity	Notes	Ref.
EV (RNA)	55 (23 GBM)	CSF	61%	98%	Compared to EGFRvIII status in tumor tissue.	[31]
EV (RNA)	73	Serum	81.6%	79.3%	Compared to EGFRvIII status in tumor tissue.	[35]
EV (miRNA)	111	CSF	67% (cisternal); 28% (lumbar)	80% (cisternal); 95% (lumbar)	9-miRNA panel compared to tumor presence.	[36]
EV (DNA)	50	Plasma	63.2%	92.6%	Compared to tumor tissue MGMT methylation.	[38]
ctDNA	35 (9 GBM)	CSF	95%	Not reported	Concordance of mutations between CSF and matched tumor tissue.	[39]
ctDNA	46	CSF	49%	Not reported	Compared to matched tumor tissue.	[40]
ctDNA	38	CSF and plasma	92,1% (CSF); 7,9% (plasma)	100% (CSF)	Compared to tumor tissue TERT promoter status.	[41]
ctDNA	4	CSF	82%	Not reported	Compared to tumor tissue; reflected key molecular alterations.	[42]
ctDNA	419 (222 GBM)	Plasma	55%	Not reported	Compared plasma ctDNA to matched tumor sequencing.	[44]
ctDNA	7 (4 GBM)	CSF and plasma	58% (CSF); 0% (plasma)	Not reported	Compared to tumor tissue. CSF better reflects mutations in CNS-restricted disease than plasma.	[45]

**Table 3:** Overview of main findings and potential clinical application of biomarkers found. Summary data based on information from [16,17,18,19,20,21,22,23,24,25,26,27,28,29,30,31,32,33,34,35,36,37,38,39,40,41,42,43,44,45,46,47,48,49,50,51,52,53,54,55,56,57,58,59,60,61,62,63,64,65,66,67,68,69,70,71,72,73,74,75,76,77,78,79].

Authors	Biomarker	Main Findings	Potential Clinical Application	Ref.
Gao et al., 2016	CTC	CTCs helped monitor treatment response and differentiate radionecrosis from glioma recurrence.	Monitoring	[16]
Lynch et al., 2020	CTC	The GLAST survey can complement GFAP probing to improve GBM-CTC identification.	Diagnosis	[17]
Bark et al., 2021	CTC	Characterization for GFAP, vimentin protein expression and *EGFR* amplification.	Diagnosis	[18]
Kolostova et al., 2021	CTC	CTCs showed high concordance with primary tumor samples and more mutations were detected.	Diagnosis and monitoring	[19]
Yang et al., 2017	Exosomes	Detection of differentially expressed genes in blood exosomes of primary and recurrent GBM; increased expression of DNM3, p65 and CD117 and decreased expression of PTEN and p53 in primary tumors; increased expression of DNM3 and p65 in recurrent tumors.	Diagnosis and prognosis	[20]
Brahmer et al., 2023	EV	EVs marker profiles were significantly increased in GBM compared to healthy controls.	Diagnosis	[25]
Dobra et al., 2020	EV, protein	sEVs were more effective in discriminating between patient groups than whole serum.	Diagnosis	[22]
Aibaidula et al., 2023	EV	Identification of a distinct phenotype (CD9+CD81+ and CD9+CD63+CD81+) and increased CD9+CD11b+CD45 phenotype of extracellular vesicles originating from nonneoplastic cells in the plasma of patients with GBM.	Diagnosis	[29]
Stella et al., 2021	EV	circSMARCA5 and circHIPK3 were significantly decreased in the sera EVs of GBM patients compared with healthy controls.	Diagnosis	[62]
Cilibrasi et al., 2022	EV	Inflammatory biomarker signature composed of several proteins (VWF, FCGBP, C3, PROS1, SERPINA1) present in EVs from GBM patients.	Diagnosis and monitoring	[23]
Hallal et al., 2020a	EV	Identification of 4054 proteins in plasma EVs. Protein profiles of EVs grouped according to glioma subtype and histological grade.	Diagnosis and monitoring	[21]
Hallal et al., 2020b	EV	miR-486-3p as well as piR_016658, 016659, and 020829 piRNAs were differentially expressed in GBM surgical aspirate and serum EVs.	Diagnosis and monitoring	[32]
Hallal et al., 2024	EV	GBM-specific proteomic signatures were determined, and putative urinary EV biomarkers corresponding to diagnosis (KRT19, RPS2, RPL18, RPL28, RPL7A), tumor burden (BCAM, ITGA3, ITM2B), and recurrence (GRN, ITM2B) were identified.	Diagnosis and monitoring	[24]
Manda et al., 2018	EV	*EGFRvIII* amplification in tumor tissues and exosomes correlated with poor survival.	Diagnosis and prognosis	[35]
Shi et al., 2015	EV	Exosomal miR-21 levels were increased in the CSF of glioma patients compared to non-glioma (brain-trauma) patients. Higher levels of tissue miR-21 are indicative of poor prognosis in the CGGA cohort.	Diagnosis and prognosis	[37]
Ricklefs et al., 2024	EV	Plasma EV concentration is increased in glioblastoma patients, and high EV levels are an independent negative prognostic parameter.	Diagnosis, prognosis and monitoring	[30]
Uziel et al., 2024	EV	h*TERT* mRNA transcript levels from EVs can be measured in serum from GBM patients. Preoperative measurements correlated with tumor volume and disease course.	Monitoring	[34]
Yekula et al., 2023	EV	Stratification of patients with recurrent GBM/*EGFR*-amplified after dacomitinib treatment; detection of a unique responder signature in the serum EV transcriptome through the biomarkers *DNMT3A*, *ZNF35*, and *LAMTOR2*.	Monitoring, patient stratification	[33]
Rosas-Alonso et al., 2024	EV	Detection of methylated *MGMT* in sEV-DNA with sensitivity and specificity of 87.5% and 90%, respectively.	Prognosis and monitoring	[38]
Lennartz et al., 2023	EV, protein	Increased Hsp70 protein levels in GBM patients associated with overall survival in different glioma subtypes.	Diagnosis and prognosis	[27]
Werner et al., 2021	EVs, protein	Elevated levels of Hsp70 showed in serum from GBM patients, suggesting its use as a tumor-specific biomarker.	Diagnosis and treatment monitoring	[28]
Zhao et al., 2020	ctDNA	Mutations in CSF ctDNA showed high concordance with tumor DNA, highlighting mutations in *RB1* and *EGFR*.	Diagnosis	[42]
Wang et al., 2015	ctDNA	Identified ctDNA in CSF from 9 GBM patients, highlighting the use of cfDNA for molecular characterization and tumor progression monitoring.	Diagnosis	[39]
Xia et al., 2021	circRNAs	Identified circRNAs (hsa_circ_0055202, hsa_circ_0074920, hsa_circ_0043722) expressed in an elevated state in GBM, validated as potential diagnostic biomarkers with high specificity.	Diagnosis	[65]
Dai et al., 2023	ctDNA	Identification of 8 differentially expressed and methylated hub genes (*COMMD1*, *C1orf226*, *CH3L2*, *FLRT2*, *ETV1*, *NKD1*, *GNB5*, and *NTRK3*) to build diagnostic and prognostic models of recurrent GBM with high accuracy.	Diagnosis and prognosis	[48]
Juratli et al., 2018	ctDNA	The *TERT*p mutation was detected in CSF ctDNA with high sensitivity and specificity and associated with patient survival.	Diagnosis and prognosis	[41]
Wang Q. et al., 2023	cfDNA	High concordance between cfDNA in CSF and tumor tissue DNA in GBM, correlating ctDNA levels with Ki67 expression.	Diagnosis and prognosis	[43]
Mattos-Arruda et al., 2015	ctDNA	CSF-derived ctDNA was more representative of brain tumor genomic alterations than plasma ctDNA, allowing identification of somatic mutations and reflecting changes in tumor burden over time.	Diagnosis and monitoring	[45]
Miller et al., 2019	ctDNA	Genomic characterization in CSF reflected tumor biopsies, allowing monitoring of glioma genome evolution. CtDNA positivity was correlated to shorter OS.	Diagnosis, monitoring and prognosis	[40]
Piccioni et al., 2019	ctDNA	Alterations in plasma cfDNA were detected in 55% of GBM patients.	Diagnosis, monitoring and stratification	[44]
Bagley et al., 2021	cfDNA	High preoperative cfDNA concentration associated with shorter progression-free survival and overall survival in patients with GBM.	Prognosis	[46]
Meng et al., 2021	cfDNA, EV, protein	MRgFUS enriches the signal of brain-derived circulating biomarkers including cfDNA, EVs, and S100b after sonication.	Diagnosis	[47]
Lucero et al., 2020	miRNA	Identification of 8 candidate miRNAs (miR-16-2-3p, miR-148a-3p, miR-182-5p, miR-9-5p, miR-9-3p, miR-22-3p, miR-186-5p, miR-378e) related to angiogenesis.	Prognosis	[49]
Akers et al., 2017	miRNA	Profiling of miRNAs derived from EVs from tumor tissue and CSF of patients with GBM, identifying a tumor signature composed of 9 miRNAs (miR-21, -218, -193b, -331, -374a, miR-548c, -520f, 27b, and 130b). Sensitivity and specificity for GBM detection were 67% and 80% for cisternal CSF, and 28% and 95% for lumbar CSF.	Diagnosis	[36]
Drusco et al., 2015	miRNA	Identification of miRNA signatures (miR-451, -711, -935, -223 and -125b) in CSF capable of distinguishing different types of brain tumors.	Diagnosis	[50]
D’Urso et al., 2015	miRNA	Increased levels of miR-21 and miR-15b in serum EVs of glioma patients, while downregulation of miR-16 distinguished GBM from low grade and anaplastic gliomas.	Diagnosis	[52]
Regazzo et al., 2016	miRNA	Decreased levels of miR-497 and miR-125b in the serum of glioma patients, with GBM having significant lower levels (AUC = 0.87 for miR-497 and 0.75 for miR-125b).	Diagnosis	[54]
Sun et al., 2015	miRNA	Significant decrease of serum miR-128 in glioma patients compared to meningioma patients and healthy controls.	Diagnosis	[53]
Lai et al., 2015	miRNA	Significantly increased miRNA-210 expression in GBM patients compared with healthy controls correlated with tumor grade and worse patient outcome.	Diagnosis and prognosis	[56]
Qu et al., 2016	miRNA	Significant increase of miR-21 in the CSF and tumor tissue of glioma patients compared to healthy controls. MiR-21 from CSF outperformed its tumor tissue counterpart as a prognostic marker.	Diagnosis and prognosis	[51]
Shao et al., 2015	miRNA	The levels of miR-454-3p in plasma in glioma patients were significantly higher than that from healthy controls (AUC= 0.9063). Increased miR-454-3p levels also correlated with higher tumor grades and poorer prognosis.	Diagnosis and prognosis	[61]
Swellam et al., 2019	miRNA	Significant increase in the levels of miR-221 and miR-222 observed in the serum of GBM patients compared to healthy controls. Higher levels of miR-221 and miR-222 were indicative of poor OS.	Diagnosis and prognosis	[60]
Xiao et al., 2016	miRNA	Significant higher levels of miR-182 in glioma patients compared to healthy controls (AUC = 0.778), specially in higher grade gliomas (AUC = 0.815). Higher miR-182 levels also correlated with shorter OS and PFS.	Diagnosis and prognosis	[55]
Yue et al., 2016	miRNA	Significant decrease of serum miR-205 in glioma patients compared to healthy individuals and other brain tumors. MiR-205 levels were inversely proportional to the tumor grade (higher grade have lower miR-205). Low miR-205 levels were an independent factor associated to poor OS.	Diagnosis and prognosis	[59]
Zhang et al., 2019	miRNA	Significantly decreased serum levels of miR-100 in GBM patients compared with healthy controls, with an increase after treatment. Low miR-100 expression was associated with unfavorable clinicopathological features and shorter survival.	Diagnosis and prognosis	[57]
Morokoff et al., 2020	miRNA	Highly accurate 9-serum miRNA signature (miR320e, miR-223, miR-16-5p, miR-484, miR520a, miR-532, miR-630, miR651, miR-761) identification in gliomas. Observed dynamic changes in specific miRNAs correlating with tumor volume over long-term follow-up.	Monitoring	[58]
Chen et al., 2020	cirRNA	Significant increase in the levels of circFOXO3, circ_0029426, and circ-SHPRH were found in the serum of GBM patients compared to healthy controls.	Diagnosis	[63]
Amer et al., 2022	lncRNA	Significant increase in lncRNA565 and lncRNA641 expression in GBM patients compared with healthy controls, correlated with clinicopathological data and unfavorable survival pattern.	Diagnosis and prognosis	[64]
Chen et al., 2017	lncRNA	High levels of serum lncRNA MALAT1 are predictive of resistance to TMZ in GBM patients. High expression of this lncRNA correlated with shorter OS and recurrence.	Prognosis and stratification	[66]
Hagemann et al., 2019	mRNA	Increased circulating *MACC1* gene transcripts in the blood of GBM patients. Elevated *MACC1* levels were associated with a worse prognosis.	Diagnosis and prognosis	[67]
Figueroa et al., 2017	mRNA, EV	Detection of DNA copy number amplification of wild-type *EGFR* and *EGFRvIII* variants. Sensitivity of 61% and specificity of 98% for detection of *EGFRvIII*-positive GBM. RNAs from EVs reflected the molecular genetic status of GBM, facilitating the guidance of specific therapies.	Diagnosis, monitoring and therapeutic decisions	[31]
Ghorbani et al., 2024	Protein	High plasma GFAP concentration was associated with GBM.	Diagnosis	[71]
Soler et al., 2017	Protein	The HLA-DR^-/low^/VNN2⁺ ratio in CD14+ PBMCs distinguishes patients with recurrent GBM from those with radiation necrosis.	Diagnosis	[70]
Tsvetkov et al., 2021	Proteins	Identified protein denaturation profiles that differentiate gliomas from healthy controls, including 19 cases of IDH wild-type GBM.	Diagnosis	[69]
Dufrusine et al., 2023	Protein, EV	Overexpression of LGALS3BP protein in EVs derived from plasma of GBM patients. Targeting extracellular LGALS3BP in xenografted mice increases survival.	Diagnosis and stratification	[26]
Masood et al., 2023	Protein, mRNA	Elevated PD-L1 levels have been associated with poor overall survival, being a potential prognostic marker and selection tool for blockade therapy.	Prognosis and stratification	[68]
Björkblom et al., 2016	Metabolite	Elevated levels of serum vitamin E (α- and γ-tocopherol) predict future GBM development years before onset.	Prediction	[72]
Bao et al., 2024	Metabolite	14 CSF metabolites were causally associated with GBM risk: 11 (α-tocopherol, butyrate, uracil, valine) linked to increased risk, and 3 (N1-methylinosine, succinylcarnitine) linked to decreased risk.	Prediction	[73]
Shen et al., 2018	Metabolite	Decreased arginine and methionine added to increased kynurenate plasma levels were associated with poor OS and PFS in newly diagnosed GBM patients.	Prognosis	[75]
Zhao et al., 2016	Metabolite	Eighteen metabolites (arginine and ornithine being the most relevant) distinguish high- from low-grade gliomas with 91.1% accuracy while six metabolites differed in quantities according to IDH mutational status.	Diagnosis, prognosis and stratification	[74]
Liu et al., 2024	Metabolite	Distinct metabolic profiles were observed in GBM tissue and patient plasma at recurrence, including N-alpha-methylhistamine, glycerol-3-phosphate, phosphocholine, and succinic acid in tissue, and indole-3-acetate and urea in plasma.	Monitoring	[76]
Bark et al., 2023	Metabolite, lipid	Detection of 151 metabolites and 197 lipids, highlighting an increase in specific metabolites in patients with unfavorable outcomes. The lipid profile showed greater heterogeneity in patients with unfavorable outcomes.	Prognosis	[77]
Zhou et al., 2022	Lipid	A panel of 11 plasma lipids was identified as serum biomarkers to distinguish malignant gliomas from healthy controls with 0.9641 accuracy. These included several phosphatidylcholines, lysophosphatidylcholines and triglycerides.	Diagnosis	[78]
Soylemez et al., 2022	Lipid	Differentially regulated lipids were identified in patients with GBM, including fatty acid, glycerolipid, glycerophospholipid, saccharolipid, sphingolipid, and sterol lipid.	Diagnosis	[79]

## Data Availability

Not applicable.

## Abbreviations

GBM	Glioblastoma
IDH	Isocitrate dehydrogenase
TMZ	Temozolomide
BBB	Blood–brain barrier
CNS	Central nervous system
CSF	Cerebrospinal fluid
EVs	Extracellular vesicles
CTCs	Circulating tumor cells
miRNA	microRNA
ctNAs	Circulating tumor nucleic acids
cfDNA	Circulating free DNA
cfRNA	Circulating free RNA
lncRNA	Long non-coding RNA
circRNA	Circular ribonucleic acid
mRNA	Messenger ribonucleic acid
piRNA	PIWI-interacting RNA
sEVs	Small extracellular vesicles
MRI	Magnetic resonance imaging
MRgFUS	Magnetic resonance imaging-guided focused ultrasound
MR	Magnetic resonance
CT	Computerized tomography
PET	Positron emission tomography
GeLB	Epigenetic Glioma Liquid Biopsy
LB	Liquid biopsy
NGS	Next-generation sequencing
n	Sample size
PFS	Progression-free survival
PBMCs	Peripheral blood mononuclear cells
GSC-EV	Glioblastoma stemcell–extracellular vesicles
qPCR	Quantitative polymerase chain reaction
ddPCR	Droplet digital polymerase chain reaction
qRT-PCR	Quantitative reverse-transcribed PCR
TERT	Telomerase reverse transcriptase
hTERT	Human telomerase reverse transcriptase
TERTp	Telomerase reverse transcriptase promoter
EGFR	Epidermal growth factor receptor
MGMT	O6-methylguanine-DNA methyltransferase
PTEN	Phosphatase and tensin homolog
GFAP	Glial fibrillar acid protein
WT	Wild-type
IHC	Immunohistochemistry
FDA	Food and drugaAdministration
WHO	World health organization
CGGA	Chinese glioma genome atlas
FFPE	Formalin-fixed paraffin-embedded
ELISA	Enzyme-linked immunosorbent assay
LC-MS	Liquid chromatography–mass spectrometry
WB	Western blot
NA	Not available
OS	Overall survival
PFS	Progression-free survival
